# Modelling of the Critical Micelle Concentration of Cationic Gemini Surfactants Using Molecular Connectivity Indices

**DOI:** 10.1007/s10953-013-0095-6

**Published:** 2013-11-02

**Authors:** Anna Mozrzymas

**Affiliations:** Department of Physics and Biophysics, Wrocław University of Environmental and Life Sciences, ul. Norwida 25, 50-375 Wrocław, Poland

**Keywords:** Cationic gemini surfactants, QSPR, Critical micelle concentration, Molecular connectivity indices

## Abstract

Modelling of the critical micelle concentrations (*cmc*) using the molecular connectivity indices was performed for a set of 21 cationic gemini surfactants with medium-length spacers. The obtained model contains only the second-order Kier and Hall molecular connectivity index. It is suggested that the index ^2^ *χ* includes some information about flexibility. The obtained model was used to predict log_10_ *cmc* of other cationic gemini surfactants. The agreement between calculated and experimental values of log_10_ *cmc* for the gemini surfactants that were not used in the correlation is very good.

## Introduction

Gemini surfactants are molecules constructed of two hydrophobic chains and two polar/ionic headgroups connected by the various spacer groups. Owing to their structure they have unique properties in aqueous solution, such as low critical micelle concentration (*cmc*) and high surface activity. The *cmc* values of these surfactants are significantly lower than those of the corresponding monomeric surfactants and in comparison to their monomeric counterparts, gemini surfactants are more efficient at reducing surface tension. Gemini surfactants demonstrate great potential for gene delivery [1]. Cationic gemini surfactants appear to be excellent for binding and compacting DNA. These surfactants bind DNA with higher efficiency and have better transfection efficiencies than their monomeric counterparts. Many conventional surfactants show good anti-microbial properties with respect to a large spectrum of bacteria, fungi and viruses, and simultaneously they are innocuous for living organisms, but the gemini compounds are much more active [2]. Due to these properties, gemini surfactants have been applied in various areas, such as the drug manufacturing especially in gene therapy, the food industry, cosmetics manufacturing especially in the skin care products, anti-bacterial and the anti-fungal preparations.

One of the main reasons for the current interest in gemini surfactants is their critical micelle concentration values which are lower, by at least one order of magnitude, than those of the corresponding monomeric surfactants. As is well known, the *cmc* depends on the molecular structure of the surfactants. In general, the *cmc* in aqueous solution decreases as the hydrophobic character of the surfactant increases. The first relationship between *cmc* and structure of a molecule was given by Klevens [3] who empirically found that logarithm of *cmc* linearly decreases with increase in hydrophobic chain length of the surfactant. Gemini surfactants have two alkyl chains and two headgroups, therefore the influence of the variation of these groups on the *cmc* can be considerable. The important factor which distinguishes gemini surfactants from conventional monomeric surfactants is the connection of the headgroups by the spacer. The nature of the spacer group (length, flexibility, chemical structure) plays an important role in regulating the aggregation properties in the solution [4].

Not long ago, a quantitative structure–property relationship (QSPR) was used for predicting the *cmc* values of conventional non-ionic [5–8] and ionic [9–12] surfactants. The values of the *cmc* of gemini surfactants can be significantly changed by a slight modification of the structure of the molecule; therefore modelling and predicting the critical micelle concentration of gemini surfactants directly from the structure of the molecule by the QSPR analysis can be of great interest. Recently, the QSPR study was performed to relate the structure of cationic gemini surfactants to their critical micelle concentration [13]. In this work, the *cmc* of gemini surfactants was correlated with 12 descriptors (seven topological among them connectivity indices, three statistical, one geometrical and one functional group descriptors).

The previous QSPR models [8, 12] show that critical micelle concentration can be correlated and predicted by using the molecular connectivity indices only. In the present work cationic gemini surfactants are taken into consideration, and just as in the previous papers, in the QSPR study ten indices are used: five connectivity indices and five valence connectivity indices, from zeroth to fourth order in both cases. These indices are calculated from the chemical structure of the molecule and they contain considerable information about the molecule, including the details of electronic structure of each atom and the molecular structure features. The information encoded in molecular connectivity indices has been demonstrated in a variety of examples [14].

As is well known the *cmc* of the surfactants depends not only on geometrical factors of the molecule but also on other parameters, such as the kind of counterion and electrostatic charge distribution; therefore, just as in the previous paper [12], in order to minimize the influence of factors other than geometrical ones, only cationic gemini surfactants with bromide as counterion were taken into account. Furthermore, among the factors significantly affecting the *cmc* in aqueous solution are the temperature of the solution and the presence in the solution of added electrolyte and various organic compounds [15]. Therefore all values of *cmc* taken in the correlation were measured in pure water at room temperature.

## Data

**Fig. 1:**
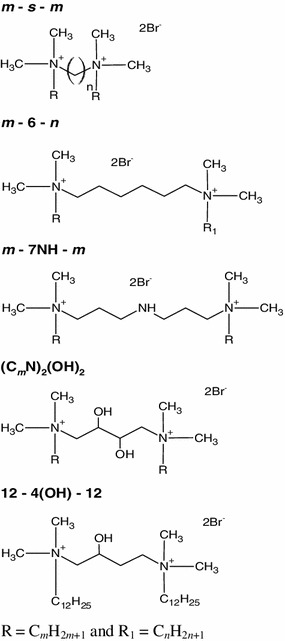
Chemical structures of the surfactants considered and their abbreviations

The data set was chosen to contain gemini surfactants with a medium-length spacer. The chemical structures of the surfactants taken into consideration and their abbreviations are shown in Fig. 1.

The *cmc* of *m*–*s*–*m* gemini surfactants [alkanediyl-α,ω-bis(dimethylalkylammonium bromide)] with a given alkyl chain, particularly for the series with *m* = 12, increases with the spacer length up to a maximum at four or five methylene units and then decrease with further increase in the number of methylene units in the spacer group [16, 17]. The *cmc* values of dissymmetric surfactants designated as *m*–6–6 are about one order of magnitude higher than those of the corresponding *m*–6–*m* symmetric surfactants [18] and the *cmc* decreases as the *m*/*n* ratio increases. In the case of the dissymmetric surfactants designated as *m*–6–*n* with *m* + *n* = 24, the *cmc* values are comparable with those of the symmetric counterparts with *m* = 12 [19] and the *cmc* slightly decreases as the *m*/*n* ratio increases. The *cmc* values of *m*–7NH–*m* (1,9-bis(dodecyl)-1,1,9,9-tetramethyl-5-imino-1,9-nonanediammonium dibromide) [20, 21] gemini surfactants are higher than those of the corresponding *m*–7–*m* gemini surfactants [20] whereas the *cmc* values of (C_*n*_N)_2_(OH)_2_ (1,4-bis(dodecyl-*N*,*N*-dimethylammonium bromide)-2,3-butanediol) [22, 23] and 12–4(OH)–12 (1,4-bis(dodecyl-*N*,*N*-dimethylammonium bromide)-2-butanol) [23] are lower than those of their hydrophobic spacer homologues. Furthermore, the *cmc* decreases with increasing hydroxyl substitution in the spacer [23].

Literature data for log_10_ *cmc* are given in Table 1. All *cmc* values were measured at 25.00 °C.

**Table 1:** The connectivity indices and the experimental $\log_{10} cmc$ values

Compound	^0^ *χ*	^1^ *χ*	^2^ *χ*	^3^ *χ*_*c*_	^4^ *χ*_*pc*_	${}^{0}\chi^{\nu }$	${}^{1}\chi^{\nu }$	${}^{2}\chi^{\nu }$	${}^{3}\chi_{c}^{\nu }$	${}^{4}\chi_{pc}^{\nu }$	log_10_ *cmc*
8–6–8	21.142	13.328	11.278	2.414	2.414	21.037	12.968	10.716	2.159	2.159	−1.292
10–6–10	23.970	15.328	12.692	2.414	2.414	23.865	14.968	12.130	2.159	2.159	−2.222
12–4–12	25.385	16.328	13.399	2.414	2.414	25.279	15.968	12.837	2.159	2.159	−2.932
12–6–12	26.799	17.328	14.107	2.414	2.414	26.693	16.968	13.544	2.159	2.159	−2.963
12–7–12	27.506	17.828	14.460	2.414	2.414	27.400	17.468	13.898	2.159	2.159	−3.046
14–6–14	29.627	19.328	15.521	2.414	2.414	29.522	18.968	14.958	2.159	2.159	−3.824
16–6–16	32.456	21.328	16.935	2.414	2.414	32.350	20.968	16.372	2.159	2.159	−4.523
16–7–16	33.163	21.828	17.289	2.414	2.414	33.057	21.468	16.726	2.159	2.159	−4.585
12–6–6	22.556	14.328	11.985	2.414	2.414	22.451	13.968	11.423	2.159	2.159	−1.790
14–6–6	23.970	15.328	12.692	2.414	2.414	23.865	14.968	12.130	2.159	2.159	−2.292
16–6–6	25.385	16.328	13.399	2.414	2.414	25.279	15.968	12.837	2.159	2.159	−2.745
13–6–11	26.799	17.328	14.107	2.414	2.414	26.693	16.968	13.544	2.159	2.159	−3.009
14–6–10	26.799	17.328	14.107	2.414	2.414	26.693	16.968	13.544	2.159	2.159	−3.022
16–6–8	26.799	17.328	14.107	2.414	2.414	26.693	16.968	13.544	2.159	2.159	−3.081
12–7NH–12	27.506	17.828	14.460	2.414	2.414	27.193	17.175	13.587	2.159	2.159	−2.932
16–7NH–16	33.163	21.828	17.289	2.414	2.414	32.850	21.175	16.415	2.159	2.159	−4.174
(C_10_N)_2_(OH)_2_	24.297	15.133	13.141	2.886	3.233	23.086	14.134	11.768	2.370	2.299	−2.432
(C_12_N)_2_(OH)_2_	27.125	17.133	14.555	2.886	3.233	25.914	16.134	13.182	2.370	2.299	−3.155
(C_14_N)_2_(OH)_2_	29.954	19.133	15.969	2.886	3.233	28.742	18.134	14.597	2.370	2.299	−4.071
(C_16_N)_2_(OH)_2_	32.782	21.133	17.384	2.886	3.233	31.571	20.134	16.011	2.370	2.299	−4.301
12–4(OH)–12	26.255	16.722	14.040	2.703	2.652	25.597	16.043	13.031	2.288	2.209	−3.027

## Methods

### Molecular Connectivity Indices (*χ*) and Valence Molecular Connectivity Indices ($\chi^{\nu }$)

Molecular connectivity indices, some of the topological descriptors to characterize molecules in structure–property and structure–activity studies, were originally proposed by Randic [24] and later developed and formalized by Kier and Hall [14]. These indices are calculated from the molecular graph, i.e. hydrogen suppressed graphic structural formula of the molecule. The molecular connectivity index is defined as

1 $$ {}^{m}\chi_{k} = \sum\limits_{j = 1}^{{n_{m} }} {\prod\limits_{i = 1}^{m + 1} {\left( {\delta_{i} } \right)_{j}^{ - 0.5} } } $$

where *m* is the order of the connectivity index, *k* denotes the type of a fragment, which is divided into paths (P), clusters (C), and path/clusters (PC). In formula 1 *n*_*m*_ is the number of relevant paths and *δ*_*i*_ is the connectivity degree and is equal to the number of atoms to which the *i*-th atom is bonded. If we replace *δ*_*i*_ by $\delta_{i}^{\nu }$, we obtain the valence molecular connectivity index ${}^{m}\chi_{k}^{\nu }$. The expression for the *m*-th order valence molecular connectivity index is as follows:

2 $$ {}^{m}\chi_{k}^{\nu } = \sum\limits_{j = 1}^{{n_{m} }} {\prod\limits_{i = 1}^{m + 1} {\left( {\delta_{i}^{\nu } } \right)_{j}^{ - 0.5} } } $$

where $\delta_{i}^{\nu }$ is the valence connectivity degree defined by

3 $$ \delta^{\nu } = \frac{{Z^{\nu } - h}}{{Z - Z^{\nu } - 1}} $$

where *Z* ^*ν*^ is the number of valence electrons in the corresponding atom, *h* is the number of hydrogen atoms connected to the *i*-th atom and *Z* is the atomic number.

An example of calculations of molecular connectivity indices for exemplary gemini surfactant and some useful information about the ^2^ *χ* index are given in Appendices A and B, respectively.

### Correlation Formula

Modelling of the critical micelle concentration as a function of molecular connectivity indices was performed for a diverse set of 21 gemini surfactants. The formula expressing the relationship between the log_10_ *cmc* and the molecular connectivity indices was generated using the least-squares method. The statistical calculations were performed using the program *STATISTICA 9.1* [25]. In the process of searching the best equation three criteria were taken into account: a correlation coefficient (*r*), a Fisher ratio value (*F*) and a standard error (*s*). The best relationship is that which has possibly highest values of *r* and *F*, and simultaneously the lowest value of *s*.

## Results and Discussion

The aim of the present work is to find the simple equation expressing the critical micelle concentration of cationic gemini surfactants as a function of the molecular connectivity indices only. In the process of searching for the simple relationship were used, just as in the previous papers [8, 12], ten indices: five molecular connectivity indices and five valence molecular connectivity indices, from zeroth to fourth order in each case. These indices were calculated for the compounds studied (Fig. 1) using Eqs. 1–3. All values of the connectivity indices and log_10_ *cmc* values are listed in Table 1.

Just as in the previous papers we started our correlation procedure with one index. This step is presented in Table 2.

**Table 2:** The values of statistical parameters for the first step

Indices	^0^ *χ*	^1^ *χ*	^2^ *χ*	^3^ *χ*_*c*_	^4^ *χ*_*pc*_	${}^{0}\chi^{\nu }$	${}^{1}\chi^{\nu }$	${}^{2}\chi^{\nu }$	${}^{3}\chi_{c}^{\nu }$	${}^{4}\chi_{pc}^{\nu }$
*r*	0.979	0.973	0.981	0.201	0.210	0.967	0.959	0.972	0.201	0.208
*F*	438.05	336.48	472.52	0.799	0.874	269.46	219.15	322.88	0.800	0.863
*s*	0.183	0.208	0.177	0.881	0.879	0.231	0.254	0.212	0.881	0.879

We see that the best correlation in this step is for the relationship containing the second-order connectivity index ^2^ *χ*, and we get the following formula:

4 $$ \log_{10} cmc = 3.971 - 0.491 \!\cdot\! {}^{2}\chi $$

Next to this index we added the remaining indices separately. The values of the correlation coefficients for second step are shown in Table 3.

**Table 3:** The values of correlation coefficients for the second step

Indices	^0^ *χ*	^1^ *χ*	^2^ *χ*	^3^ *χ*_*c*_	^4^ *χ*_*pc*_	${}^{0}\chi^{\nu }$	${}^{1}\chi^{\nu }$	${}^{2}\chi^{\nu }$	${}^{3}\chi_{c}^{\nu }$	${}^{4}\chi_{pc}^{\nu }$
*r*	0.981	0.981	**–**	0.981	0.981	0.981	0.981	0.982	0.981	0.981

The addition of other indices in the second step did not change significantly the correlation coefficient and other parameters; therefore at first step the process of searching for the best relationship was ended.

**Fig. 2:**
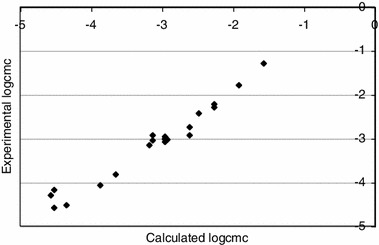
Scatter plot of the calculated log_10_ *cmc* versus the experimental log_10_ *cmc* (*r* = 0.981, *F* = 472.52, *s* = 0.177)

The comparison between the experimental values of log_10_ *cmc* with those calculated from Eq. 4 is shown in Fig. 2.

The calculated values of log_10_ *cmc* using the obtained model (Eq. 4), along with the experimental values of log_10_ *cmc* for the surfactants studied, are given in Table 4.

**Table 4:** Calculated and literature values of log_10_ *cmc* for the studied gemini surfactants

Compound	Calculated log_10_ *cmc*	Experimental log_10_ *cmc*
8–6–8	−1.566	−1.292
10–6–10	−2.261	−2.222
12–4–12	−2.608	−2.932
12–6–12	−2.956	−2.963
12–7–12	−3.129	−3.046
14–6–14	−3.650	−3.824
16–6–16	−4.344	−4.523
16–7–16	−4.518	−4.585
12–6–6	−1.914	−1.790
14–6–6	−2.261	−2.292
16–6–6	−2.608	−2.745
13–6–11	−2.956	−3.009
14–6–10	−2.956	−3.022
16–6–8	−2.956	−3.081
12–7NH–12	−3.129	−2.932
16–7NH–16	−4.518	−4.174
(C_10_N)_2_(OH)_2_	−2.481	−2.432
(C_12_N)_2_(OH)_2_	−3.176	−3.155
(C_14_N)_2_(OH)_2_	−3.870	−4.071
(C_16_N)_2_(OH)_2_	−4.565	−4.301
12–4(OH)–12	−2.923	−3.027

From Table 4 it follows that the calculated values of log_10_ *cmc* are very close to the experimental ones.

Inspection of the data in Tables 1 and 4 reveals that, in agreement with the experiments, as the length of the alkyl chains increase and in consequence the values of index ^2^ *χ* increase then the *cmc* decreases. For example, for the compounds *m*–6–*m* with *m* = 8, 10, 12, 14, 16 we obtain the following values of index ^2^ *χ*: 11.278, 12.692, 14.107, 15.521, 16.935 and the following calculated values of *cmc*: 27.13, 5.49, 1.11, 0.22, 0.05 (mmol·L^−1^), and the experimental values of *cmc* are the following: 51, 6, 1.09, 0.15, 0.03 (mmol·L^−1^) [16, 17, 26], and for the compounds (C_*m*_N)_2_(OH)_2_ with *m* = 10, 12, 14, 16 we obtain the following values of index ^2^ *χ*: 13.141, 14.555, 15.969, 17.384 and calculated values of *cmc*: 3.30, 0.67, 0.14, 0.03 (mmol·L^−1^), and the experimental values of *cmc* are the following: 3.7, 0.7, 0.085, 0.05 (mmol·L^−1^) [22], respectively. For the compounds with imino-substituted spacer group, a decrease in the calculated values of *cmc* with increasing alkyl chain length is also observed. Next, when the number of methylene groups increases in the spacer group and in consequence the values of index ^2^ *χ* increase, then the experimental and also the calculated values of *cmc* decrease. For example, for the compounds 12–*s*–12 with *s* = 4, 6, 7 we obtain the following values of index ^2^ *χ*: 13.399, 14.107, 14.460 and calculated values of *cmc*: 2.47, 1.11, 0.74 (mmol·L^−1^) and the experimental values of *cmc* are the following: 1.17, 1.09, 0.9 (mmol·L^−1^) [17, 20], respectively. From Table 4 we can also see that both the experimental and calculated values of *cmc* decrease with increasing hydroxyl substitution in the spacer and the values of index ^2^ *χ* increase also. For example, for the compounds 12–4(OH)_*n*_–12 with *n* = 0, 1, 2 we obtain the following values of index ^2^ *χ*: 13.399, 14.04, 14.555 and calculated values of *cmc*: 2.47, 1.20, 0.67 (mmol·L^−1^) and the experimental values of *cmc* are the following: 1.17, 0.94, 0.7 (mmol·L^−1^) [17, 22, 23], respectively.

If we take into account only the spacer group we can see that for given gemini surfactants the experimental values of *cmc* decrease with increasing number of methylene groups or hydroxyl substitution in the spacer. For 12–*s*–12 gemini surfactants, as the spacer increases in length it becomes more flexible [17]. In the case of 12–4(OH)_*n*_–12 gemini surfactants, the increase in hydroxyl substitutions in the spacer group may also cause the increase in the flexibility of that group [22]. From the obtained relationship (Eq. 4) it follows that when the number of atoms and/or the number of branches in the spacer group increase, and in consequence the value of ^2^ *χ* increases then the *cmc* decreases. This may suggest that the index ^2^ *χ* includes some information about the flexibility of that group.

The obtained model was used to predict log_10_ *cmc* for some other cationic gemini surfactants to test Eq. 4; the results are shown in Table 5.

**Table 5:** Test of Eq. 4

Compound	Calculated log_10_ *cmc*	Experimental log_10_ *cmc*	Ref.
	−3.087	−3.013	[21]
	−2.997	−2.951	[27]
	−3.444	−3.409	[28]

As shown in Table 5, the agreement between calculated and experimental values of log_10_ *cmc* for the cationic gemini surfactants which were not used in the correlation is very good.

The data contained in Tables 4, 5 confirm the conclusion that when the number of branches in the spacer group increases then the critical micelle concentration decreases. For example, for the compounds with six atoms in the spacer group: 12–6–12, 12–5N–12 [21] and (C_12_N)_2_(OH)_2_, we obtain the following values of index ^2^ *χ*: 14.107, 14.375, 14.555, the following calculated values of *cmc*: 1.11, 0.82, 0.67 (mmol·L^−1^), and the experimental values of *cmc* are the following: 1.09, 0.97, 0.7 (mmol·L^−1^) [17, 21, 22], respectively.

## Conclusion

From the obtained relationship, it follows that when the number of atoms and/or the number of branches increase in the spacer group then the *cmc* decreases. This refers not only to the spacer group but also to the whole molecule and is in agreement with some experimental and also theoretical results obtained for conventional surfactants [10, 15]. The increase in the number of atoms or branches influences the flexibility and consequently micelle formation. This suggests that the ^2^ *χ* index, appearing in the model, includes some information about flexibility.

The results obtained for the compounds taken into consideration (Tables 4, 5) show that the obtained model, which contains only the Kier and Hall index of second-order, can be used to predict the *cmc* of cationic gemini surfactants especially bis-quaternary ammonium bromide salts with medium-length spacers and can be helpful in designing novel cationic gemini surfactants.

## Figures and Tables

**Fig. 3:**
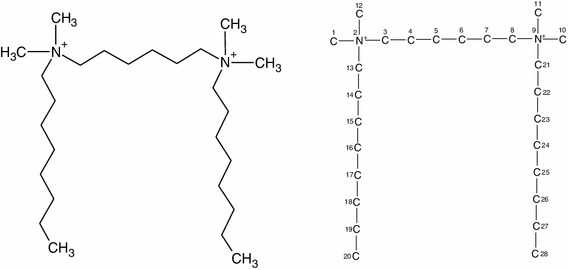
The molecular structure and the molecular graph of 8–6–8 gemini surfactant

**Fig. 4:**
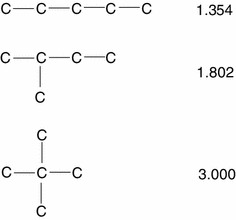
The molecular graphs of the isomers of pentane and corresponding values of index ^2^ *χ*

**Fig. 5:**
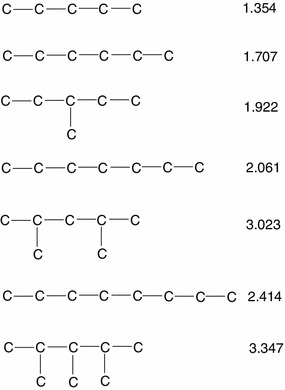
The comparison of values of index ^2^ *χ* for the molecular graphs with different number of atoms

## Appendix A

### Example of Calculations of the Indices

This section illustrates the calculations of molecular connectivity and valence molecular connectivity indices for the 8–6–8 gemini surfactant. These indices are calculated from the molecular graph in which vertices represent atoms and edges symbolize covalent bonds. The molecular structure and the corresponding molecular graph of the compound are shown in Fig. 3.

The numbers 1–28 are the labels of the atoms in the graph. The corresponding values of connectivity degree are shown in Table 6.

**Table 6:** The values of $\delta_{i}$ and $\delta_{i}^{\nu }$

Labels of atoms	1	2	3	4	5	6	7	8	9	10	11	12	13	14	15	16	17	18	19	20	21	22	23	24	25	26	27	28
*δ*	1	4	2	2	2	2	2	2	4	1	1	1	2	2	2	2	2	2	2	1	2	2	2	2	2	2	2	1
*δ* ^*ν*^	1	5	2	2	2	2	2	2	5	1	1	1	2	2	2	2	2	2	2	1	2	2	2	2	2	2	2	1

The calculations of connectivity indices (from zeroth to fourth order) and valence connectivity indices (from zeroth to fourth order) are the following:

$$ \begin{aligned} &{}^{0}\chi = \sum {\left( {\delta_{i} } \right)^{ - 0.5} = 20 \cdot 2^{ - 0.5} + 6 \cdot 1^{ - 0.5} + 2 \cdot 4^{ - 0.5} = 21.142}\\ & {}^{1}\chi = \sum {\left( {\delta_{i} \times \delta_{j} } \right)^{ - 0.5} } = 2 \cdot (1 \times 2)^{ - 0.5} + 17 \cdot (2 \times 2)^{ - 0.5} + 4 \cdot (2 \times 4)^{ - 0.5} + 4 \cdot (1 \times 4)^{ - 0.5} = 13.328  \\ & {}^{2}\chi = \sum {\left( {\delta_{i} \times \delta_{j} \times \delta_{k} } \right)^{ - 0.5} = 2} \cdot (1 \times 2 \times 2)^{ - 0.5} + 14 \cdot (2 \times 2 \times 2)^{ - 0.5} + 6 \cdot (2 \times 2 \times 4)^{ - 0.5} + 8 \cdot (2 \times 4 \times 1)^{ - 0.5}\\ & \quad \quad \quad + 2 \cdot (1 \times 4 \times 1)^{ - 0.5} = 11.278\\ & {}^{3}\chi_{c}  = \sum {\left( {\delta_{i} \times \delta_{j} \times \delta_{k} \times \delta_{l} } \right)^{ - 0.5} = 4 \cdot (1 \times 4 \times 2 \times 1)^{ - 0.5} + 4 \cdot (1 \times 4 \times 2 \times 2)^{ - 0.5} = 2.414}\\ & {}^{4}\chi_{pc} = \sum {\left( {\delta_{i} \times \delta_{j} \times \delta_{k} \times \delta_{l} \times \delta_{m} } \right)^{ - 0.5} = 4 \cdot (1 \times 4 \times 1 \times 2 \times 2)^{ - 0.5} + 8 \cdot (1 \times 4 \times 2 \times 2 \times 2)^{ - 0.5} = 2.414}\\ \end{aligned} $$

and

$$ \begin{aligned}&{}^{0}\chi^{\nu } = \sum {\left( {\delta_{i}^{\nu } } \right)^{ - 0.5} = 20 \cdot 2^{ - 0.5} + 6 \cdot 1^{ - 0.5} + 2 \cdot 5^{ - 0.5} = 21.037}  \\ &{}^{1}\chi^{\nu } = \sum {\left( {\delta_{i}^{\nu } \times \delta_{j}^{\nu } } \right)^{ - 0.5} } = 2 \cdot (1 \times 2)^{ - 0.5} + 17 \cdot (2 \times 2)^{ - 0.5} + 4 \cdot (2 \times 5)^{ - 0.5} + 4 \cdot (1 \times 5)^{ - 0.5} = 12.968  \\ & {}^{2}\chi^{\nu } = \sum {\left( {\delta_{i}^{\nu } \times \delta_{j}^{\nu } \times \delta_{k}^{\nu } } \right)^{ - 0.5} = 2 \cdot (1 \times 2 \times 2)^{ - 0.5} + 14 \cdot (2 \times 2 \times 2)^{ - 0.5} + 6 \cdot (2 \times 2 \times 5)^{ - 0.5} + 8 \cdot (2 \times 5 \times 1)^{ - 0.5} }  \\ & \quad \quad \quad + 2 \cdot (1 \times 5 \times 1)^{ - 0.5} = 10.716  \\ & {}^{3}\chi_{c}^{\nu } = \sum {\left( {\delta_{i}^{\nu } \times \delta_{j}^{\nu } \times \delta_{k}^{\nu } \times \delta_{l} } \right)^{ - 0.5} = 4 \cdot (1 \times 5 \times 2 \times 1)^{ - 0.5} + 4 \cdot (1 \times 5 \times 2 \times 2)^{ - 0.5} } = 2.159  \\ & {}^{4}\chi_{pc}^{\nu } = \sum {\left( {\delta_{i}^{\nu } \times \delta_{j}^{\nu } \times \delta_{k}^{\nu } \times \delta_{l}^{\nu } \times \delta_{k}^{\nu } } \right)^{ - 0.5}} =  4 \cdot (1 \times 5 \times 1 \times 2 \times 2)^{ - 0.5} + 8 \cdot (1 \times 5 \times 2 \times 2 \times 2)^{ - 0.5} = 2.159  \\ \end{aligned} $$

## Appendix B

### Information About the ^2^*χ* Index

The second order connectivity index ^2^ *χ* appearing in the obtained model does not differentiate heteroatoms, it includes information about three-atom fragments and its values depend on the isomers of the compound, the values of ^2^ *χ* increase with increased branching in the molecule [14]. The dependence of the values of index ^2^ *χ* on the isomers of pentane is shown in Fig. 4.

The molecular graphs of the isomers of pentane are ranked according to the increasing values of index ^2^ *χ*. We can see that the increase in branching in the molecule results in the increase the values of index ^2^ *χ*. It is also evident that when the number of atoms increases in the molecule then ^2^ *χ* also increases. But if the increase the number of atoms in the molecule is related to increasing of branches, then the increase in ^2^ *χ* is larger. The influence of the number of atoms in the molecule on the values of index ^2^ *χ* is presented in Fig. 5.
